# Epidemiological assessment of predictors of caries increment in 7-10-year-olds: a 2-year cohort study

**DOI:** 10.1590/S1678-77572010000200004

**Published:** 2010

**Authors:** Ariana Bellotto Correa KASSAWARA, Elaine Pereira da Silva TAGLIAFERRO, Karine Laura CORTELLAZZI, Gláucia Maria Bovi AMBROSANO, Andréa Videira ASSAF, Marcelo de Castro MENEGHIM, Antonio Carlos PEREIRA

**Affiliations:** 1 DDS, MSc, PhD, Department of Community Dentistry, Piracicaba Dental School, State University of Campinas, Piracicaba, SP, Brazil.; 2 DDS, MSc, PhD, Department of Community Dentistry, Piracicaba Dental School, State University of Campinas, Piracicaba, SP, Brazil.; 3 Agr. Eng., MSc, PhD, Full Professor at Department of Community Dentistry, Piracicaba Dental School, State University of Campinas, Piracicaba, SP, Brazil.; 4 DDS, MSc, PhD, Associate Professor at Department of Community Dentistry, Piracicaba Dental School, State University of Campinas, Piracicaba, SP, Brazil.; 5 DDS, MSc, PhD, Full Professor at Department of Community Dentistry, Piracicaba Dental School, State University of Campinas, Piracicaba, SP, Brazil.; 6 DDS, MSc, PhD, Department of Community Services, Dental School, Fluminense Federal University, Niterói, RJ, Brazil.

**Keywords:** Dental caries, Epidemiology, Risks prediction

## Abstract

**Objective:**

The aim of this 2-year cohort study (2003 to 2005) was to investigate how caries experience, at initial lesions (early or non-cavited lesions) and cavited stages, predicts caries increment in permanent teeth in 7-10- year-olds.

**Material and Methods:**

The random sample of 765 children attending public schools in the city of Piracicaba, SP, Brazil, was divided into two groups: 423 children aged 7-8 years and 342 children aged 9-10 years. All subjects were examined by a calibrated examiner, using dental mirror and ball-ended probes, after tooth brushing and air-drying in an outdoor setting, based on the World Health Organization criteria. Active caries with intact surfaces were also recorded as initial lesion (IL). Univariate analysis was used for statistical analysis (Odds Ratios and Chisquare).

**Results:**

The association between the DMFT (decayed, missing and filled teeth) increment and the presence of IL was significant only for 9-10-year-old children. The children with DMFT>0 at baseline were more prone to have DMFT increment, with the highest risk for caries increment occurring in children aged 7-8 years.

**Conclusion:**

The predictors of caries increment were the presence (at baseline) of caries experience in permanent teeth for both age groups (7-8; 9-10-year-olds) and the presence of the IL (at baseline) for 9-10-year-olds.

## INTRODUCTION

Several epidemiological studies have discussed the changes in dental caries diagnosis criteria^6,7,18^.

Evidence from literature has shown that the early detection of initial caries lesions and the preventive approach are both the main aims for maintaining a good oral health status^2,6,7,14^. In fact, detecting initial caries lesions in epidemiological studies is important to estimate the real disease prevalence and to know the treatment needs, targeting either invasive or non invasive procedures, to subjects and/or groups of populations at risk.

Since recent scientific studies have reported that initial caries lesions are significantly more prevalent than cavitated caries lesions^7^, it is also important to determine the impact of initial caries lesions in caries risk assessment, verifying its influence as a predictor of caries increment, as assessed by some studies^8,20,23^. Therefore, the aim of this cohort study was to investigate how caries experience, at initial caries lesions (early lesions) and cavitated stages, predicts caries increment in permanent teeth over a two-year period.

## MATERIAL AND METHODS

### Ethical Aspects

The study was approved by the Research Ethics Committee of Piracicaba Dental School, State University of Campinas (FOP/UNICAMP), under the protocol number 151/2003. The School Principals granted permission for the study and an informed consent was obtained from each child’s parents. All children diagnosed with treatment needs in the epidemiological examinations were treated at FOP/UNICAMP’s preventive-restorative program.

### Study Design

This is a two-year prospective cohort study conducted between 2003 and 2005 in schoolchildren attending four different schools in the city of Piracicaba, SP, Brazil. At baseline (2003), 983 7-10-year-old schoolchildren of both genders were examined for dental caries and 765 children (77.8% response rate) were reexamined in 2005 (final examination).

### Study Location

Piracicaba is a city located in the State of São Paulo in the southern region of Brazil. Its population is about 365,000 inhabitants, and its Human Development Index is 0.81. Fluoride has been added to the water supply system since 1971. Over the last 3 decades, epidemiological surveys have shown that caries disease has decreased significantly, mainly due to the fluoridated water supply and use of fluoridated dentifrice by the population.^16^

### Sample

In order to calculate the sample size, the random sampling method was used considering the caries experience by age group, based on previous surveys carried out in Piracicaba with a design error of 2, sample error 2%, a sampling loss of 20% and a confidence level of 95%, that added up to 1,037 children. Considering the exclusion criteria: having no parental consent, presence of systemic diseases or communication and/or neuromuscular disorders, use of a fixed orthodontic appliance, presence of severe fluorosis or hypoplasia (n=54), the initial sample was composed of 983, 7 to 10-year-old children. In 2005, 765 children aged 9 to 12 years were reexamined for dental caries.

All the schools selected in this study are run by the municipality and are situated in low-income urban communities from the outskirts of Piracicaba. The schoolchildren were similar concerning socioeconomic characteristics.

### Examination Methodology

The dental examinations carried out in 2003 (baseline) and in 2005 (final examination) followed the same protocol. All subjects were examined by a calibrated examiner, helped by a note-taking assistant, using dental mirror and ball-ended probes with a diameter of 0.5 mm for removing debris and assessing presence of fissure sealants in case of doubt and also to check the surface texture of initial lesion (IL). Before the examination each child brushed their teeth with fluoridated dentifrice for about 2 min, using the modified Bass technique, under supervision of a dental hygienist. Moreover, dental drying was carried out for about 5 s per tooth using air from a dental compressor (Proquest Delivery System, model 4010, Compressor Technologies LTD, Englewood, USA). Examinations were performed only on days with an appropriate natural luminosity with the child seated in a school chair in front of the examiner. No radiographs were taken in both baseline and final examinations.

### Diagnostic Thresholds, Criteria and Codes

The criteria and codes used in this study were those based on the WHO recommendations^25^ that consider a tooth as decayed only when cavitations are present. Active caries with intact surfaces were also recorded as ILs, following an adaptation of the criteria proposed by Nyvad, et al.^15^ (1999) and Fyffe, et al.^5^ (2000). Thus, an IL is defined as a presumably active carious lesion which, upon visual assessment by a calibrated examiner, has an intact surface with no clinically detectable dental tissue loss, with a whitish/yellowish area of increased opacity, roughness and loss of luster. When the probe is used, its tip should move gently across the surface. For the smooth surfaces, caries lesion is typically located close to gingival margin. For the occlusal surface, the lesion extends along the fissure walls. In this study, localized surface defects (active microcavities) restricted to enamel were also included in the IL group. IL and microcavities contiguous to sealants, restorations and cavitations were also recorded.

Two diagnostic thresholds were used in the study: WHO diagnostic thresholds (DMFT decayed, missing and filled permanent teeth index) and WHO+IL diagnostic threshold (DMFT index + initial caries active lesions).

### Calibration of the Examiner

One examiner with epidemiological experience in surveys using the World Health Organization criteria^25^ was calibrated before baseline and final examinations by a benchmark “Gold Standard” dental examiner, who routinely uses the WHO criteria for training and calibration for oral health surveys. The benchmark examiner had also been previously trained and calibrated in diagnosing ILs using similar criteria of other studies^2,3^. The calibration process consisted of theoretical discussions in classrooms and clinical training sessions held in outdoor setting, under natural light, in four periods of 4 h. Mean inter-examiner agreement (benchmark examiner versus examiner), measured by the Kappa statistics^9^ was K=0.88 for the WHO+IL. Reexaminations were performed in 10% of the sample to determine intra-examiner error. Mean weighted Kappa value of intra-examiner agreement was K=0.89 (WHO+IL).

### Data Analysis

In data analysis the dependent variable was DMFT increment>0 over the 2-year period (DMFT at final examination subtracted from DMFT at baseline, according to the WHO). First the influence of ILs on caries increment was tested according to age groups (7-8-year-olds and 910-year-olds) by the Chi-square test. Then each age group was divided according to caries experience as follows: DMFT and dmft=0 (Control group); DMFT=0 and dmft>0 (caries experience in primary teeth); DMFT>0 and dmft>0 (caries experience in permanent and caries experience or no in primary teeth) to test the influence of caries experience at cavitated stage on the caries increment. Odds Ratios (OR), their 95% confidence intervals (CI) and significance levels were estimated. All statistical tests were performed using the software SAS^19^ at 5% significance level.

## RESULTS

The response rate in this 2-year cohort study was 77.8%, as from the 983 children who were examined at baseline, 765 completed the study. The reasons for children dropout were: moving to another school and refusal to participate in the final examination.

Table 1 shows the results of the univariate analyses where the association between DMFT increment (dependent variable) and age groups considering the presence or absence of ILs was assessed. The presence of IL at baseline affected the caries increment in permanent dentition after 2 years only for 9-10-year-old children. Among those with ILs (n=62), 37.1% (n=23) presented DMFT increment after 2 years.

**Table 1:** Decayed missing and filled teeth (DMFT) increment according to the presence/absence of initial lesions (OR: odds ratio; CI: confidence interval)

**Age group**	**Initial lesion**	**DMFT Increment>0**		**OR**	**95%CI**	***p* -value**
		** Number of children**	**%**			
7-8-year-olds	No	93	27.0	1.00		
	Yes	24	30.8	1.20	0.70-2.06	0.497
9-10-year-olds	No	69	24.6	1.00		
	Yes	23	37.1	1.80	1.00-3.23	0.045

Table 2 shows that for both age groups children with DMFT>0 (not included ILs) were more prone to have DMFT increment, with the highest risk for caries increment occurring in children aged 7-8 years.

**Table 2:** Decayed missing and filled teeth (DMFT) increment according to caries experience at baseline (OR: odds ratio; CI: confidence interval)

**Groups**	**DMFT Increment>0**		**OR**	**95%CI**	***p* -value**
	**Number of children**	**%**			
7-8-year-olds with DMFT and dmft=0	30	19.0	1.00		
7-8-year-olds with DMFT=0 and dmft>0	55	29.1	1.25	0.82-1.91	0.302
7-8-year-olds with DMFT>0 and dmft>0	32	42.1	9.87	4.26-22.78	<0.001
9-10-year-olds with DMFT and dmft=0	30	22.1	1.00		
9-10-year-olds with DMFT=0 and dmft>0	21	23.6	1.08	0.60-1.94	0.789
9-10-year-olds with DMFT>0 and dmft>0	41	35.0	2.96	1.51-5.78	0.002

## DISCUSSION

In this prospective cohort study investigating how caries experience predicts caries increment in permanent teeth, the presence of IL at baseline was associated with caries increment in permanent dentition after 2 years for 9-10-yearold children (Table 1). This indicates that this variable is an important clinical finding and that these children should be assisted regularly.

Another important result is that all the children with DMFT>0 and dfmt>0 were significantly more prone to develop caries in permanent dentition in comparison to those caries-free in both dentitions (Table 2). The children aged 7-8 years were probably in a dentition transition phase, with eruption of several permanent teeth, especially the first molars and incisors, when data were collected. As pointed out by Carvalho et al.^4^ (1989), teeth in eruption process are more susceptible to develop dental caries because the biofilm finds favorable conditions for accumulation as these teeth are not yet in function. Moreover, children may present poor dental cleaning, becoming a more susceptible group for dental caries in permanent dentition.

An unexpected result was that a child with caries experience in permanent dentition at baseline presented statistically higher probability of developing DMFT increment (Table 2), since many studies about caries prediction have shown that caries in primary dentition is a good predictor of the disease in permanent dentition^10-12,21,22,24^. In general, children aged 7-8 years are in a period of dentition transition, with many primary teeth exfoliating and permanent teeth in eruption. Especially the last condition may increase the caries risk due to some characteristics such as: a) a higher carbonate content in dental enamel, which causes changes in the hydroxyapatite crystal lattice, resulting in a more acid-susceptible enamel surface; and b) teeth in eruption have no functional occlusal contact, which may increase dental biofilm accumulation and hinder toothbrushing^1^.

Regarding the lack of association between the DMFT increment and the presence of ILs for younger children (Table 2), in a study of Neilson and Pitts^13^ (1991), 74% of ILs detected at baseline halted or receded during the 2-year period of the research. It is also important to note that in a recent study also conducted in Piracicaba with 6-8 year-olds, there was no association between ILs and caries increment after 7 years of evaluation.^21^ However, the inclusion of ILs within epidemiological surveys is likely to establish a clearer relation between the epidemiological estimates of dental caries prevalence and the treatment needs.^17^ Moreover, in a longitudinal study, changes in behavioral characteristics, such as, improvement in dental cleaning, reduction in sugar consumption, etc., may modify the oral environment in such a way that caries risk declines over time. Therefore data collection in relation to ILs in oral health surveys should be encouraged in order to know the real prevalence of caries and to help oral health administrators in planning prevention actions.

It is also important to mention the limitations of this study. The results cannot be extrapolated to all populations and regions or countries because some differences exist compared to other regions, such as availability of fluoridated dentifrice since about 1990’s and water fluoridation since 1971.

## CONCLUSION

The predictors of caries increment were the presence (at baseline) of caries experience in permanent teeth for both age groups (7-8; 910-year-olds) and the presence of the IL (at baseline) for 9-10-year-olds.
